# Tetrahedral framework nucleic acids promote scarless healing of cutaneous wounds via the AKT-signaling pathway

**DOI:** 10.1038/s41392-020-0173-3

**Published:** 2020-07-17

**Authors:** Junyao Zhu, Mei Zhang, Yang Gao, Xin Qin, Tianxu Zhang, Weitong Cui, Chenchen Mao, Dexuan Xiao, Yunfeng Lin

**Affiliations:** State Key Laboratory of Oral Diseases, National Clinical Research Center for Oral Diseases, West China Hospital of Stomatology, Sichuan University, 610041 Chengdu, P.R. China

**Keywords:** Tissue engineering, Stem cells

## Abstract

While the skin is considered the first line of defense in the human body, there are some vulnerabilities that render it susceptible to certain threats, which is an issue that is recognized by both patients and doctors. Cutaneous wound healing is a series of complex processes that involve many types of cells, such as fibroblasts and keratinocytes. This study showed that tetrahedral framework nucleic acids (tFNAs), a type of self-assembled nucleic-acid material, have the ability to promote keratinocyte(HaCaT cell line) and fibroblast(HSF cell line) proliferation and migration in vitro. In addition, tFNAs increased the secretion of vascular endothelial growth factor (VEGF) and basic fibroblast growth factor (bFGF) in HSF cells and reduced the production of tumor necrosis factor-alpha (TNF-α) and interleukin-1 beta (IL-1β) in HaCaT cells by activating the AKT-signaling pathway. During in vivo experiments, tFNA treatments accelerated the healing process in skin wounds and decreased the development of scars, compared with the control treatment that did not use tFNAs. This is the first study to demonstrate that nanophase materials with the biological features of nucleic acids accelerate the healing of cutaneous wounds and reduce scarring, which indicates the potential application of tFNAs in skin tissue regeneration.

## Graphical abstract

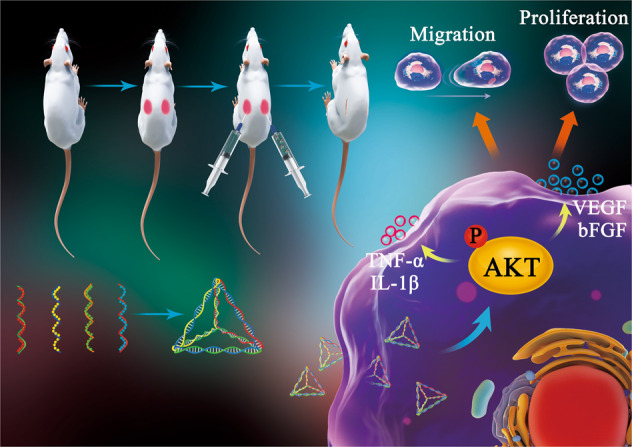

## Introduction

The skin forms the first line of defense in the human body.^1^ However, due to its fragility and location, it can be damaged as a result of injury or surgery. The wound healing process in the skin involves a series of complex phases including three major stages: the inflammation phase, the tissue formation phase and the tissue reorganization and remodeling phase.^2,3^ The inflammation phase involves the migration of macrophages and phagocytic neutrophils to the wound areas. During this phase, the release of inflammatory cytokines promotes both the migration and proliferation of fibroblasts.^3^ The tissue formation phase includes the formation of collagen deposits, granulation tissue, and epithelial metaplasia.^3^ The tissue reorganization and remodeling phase involves collagen remodeling and tissue formation to create a scar.^3,4^ Poorly healed wounds often leave behind prominent scars, which can be detrimental to a patient’s physical and mental well-being. Therefore, methods to accelerate wound healing and reduce scarring are the main focus of recent research in this field. To reduce scarring, it is necessary to control inflammatory reactions, increase fibroblast migration and proliferation, and promote epithelialization during wound healing.^3^

Scientists have recently begun concentrating on stem cell and gene therapies as strategies to bolster the wound healing process.^5–8^ Unfortunately, stem cells are difficult to isolate and culture.^5,7^ In addition, gene therapy, which utilizes various types of mRNA with the help of delivery substances, can be wasteful and inefficient.^6^ Therefore, a material that is easy to source and can modulate the various stages of skin wound healing may be a potential solution to accelerate this process and to reduce the formation of scar tissue.

Tissue regeneration is an ongoing challenge for scientists.^9,10^ Nanotechnology has been applied in different biomedical fields with some success.^11–17^ However, this platform still faces many challenges, especially with regard to tissue regeneration.^18,19^ Tetrahedral framework nucleic acids (tFNAs) are self-assembled nucleic-acid materials that can be easily synthesized and used,^20^ and they are of favorable safety owing to the biological nature of nucleic acids.^21^ Self-assembled tFNAs comprise four single-stranded DNAs (ssDNAs) based on complementary base pairings.^22,23^ In contrast to regular ssDNAs, which are difficult to incorporate into cells, tFNAs can be taken up in abundance through caveolin-mediated endocytosis without any further delivery assistance.^24–26^ Previous studies have demonstrated that tFNAs have the capacity to provoke cell proliferation and migration and to reduce inflammatory reactions.^27–30^ In addition, tFNAs may influence different signaling pathways, such as the Wnt pathway and the Nrf2 pathway.^27,28^ However, to the best of our knowledge, there have been few reports regarding nucleic-acid nanophase materials that directly affect the skin wound healing process without delivery assistance. In this study, we focused on evaluating the effects of tFNAs on keratinocytes (HaCaT cell line) and fibroblasts(HSF cell line) in vitro and in rat wound models in vivo.

## Results

### Characterization of tFNAs and cell uptake

**Fig. 1:**
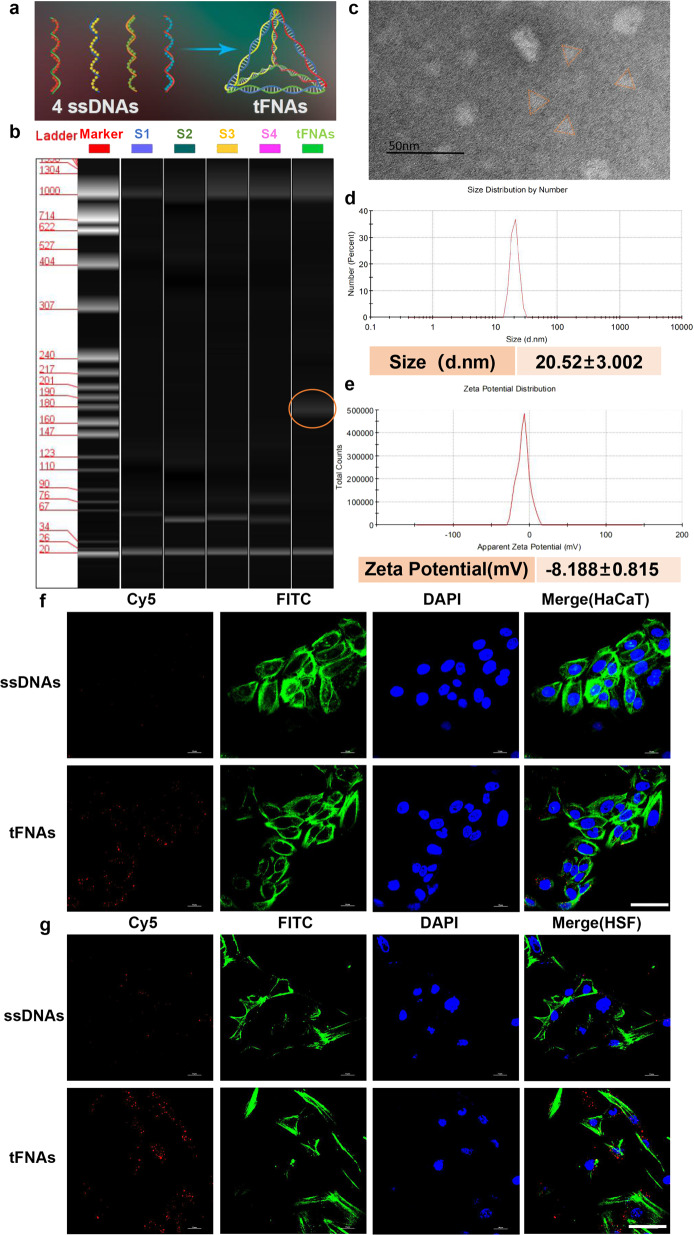
Characteristics and cellular uptake of tFNAs. **a** Structure of tFNAs. **b** Results of HPCE showing the successful assembly of tFNAs. **c** A TEM image showing the tFNA morphology. **d** Size of the tFNA molecule. **e** Zeta potential of the synthesized tFNAs. **f** Cy5-ssDNAs and Cy5-tFNAs taken up by HaCaT cells (Cy5-ssDNAs and Cy5-tFNAs: red; nucleus: blue; cytoskeleton:green). Scale bars are 100 pm. **g** Cy5-ssDNAs and Cy5-tFNAs taken up by HSF cells (Cy5-ssDNAs and Cy5-tFNAs: red; nucleus: blue; cytoskeleton:green). Scale bars are 100 pm

tFNAs contain four ssDNA molecules that orient to form a tetrahedral framework via specific base pairing (Fig. 1a). In this study, we used the same ssDNAs as those in our former studies (Table 1).^25–33^ The high-performance capillary electrophoresis(HPCE) results showed that one tFNA molecule was composed of four ssDNA molecules (Fig. 1b). Transmission electron microscopy(TEM) was used to examine the geometrical structure of tFNAs, and triangle-shaped structures were observed (Fig. 1c). In addition, we analyzed the size of the tFNA molecule; the average size was 20.52 ± 3.002 nm (Fig. 1d). The results of the zeta potential measurement of tFNAs indicated negatively charged surfaces of −8.188 ± 0.815 mV (Fig. 1e), which suggested their stability in TM buffer(10 mM Tris-HCl, 50 mM MgCl2, pH = 8.0) solution.

**Table 1:** Base sequences of the ssDNAs used to construct the tFNAs

ssDNA	Direction	Base sequence
S1	5′→3′	ATTTATCACCCGCCATAGTAGACGTATCACCAGGCAGTTGAGACGAACATTCCTAAGTCTGAA
S2	5′→3′	ACATGCGAGGGTCCAATACCGACGATTACAGCTTGCTACACGATTCAGACTTAGGAATGTTCG
S3	5′→3′	ACTACTATGGCGGGTGATAAAACGTGTAGCAAGCTGTAATCGACGGGAAGAGCATGCCCATCC
S4	5′→3′	ACGGTATTGGACCCTCGCATGACTCAACTGCCTGGTGATACGAGGATGGGCATGCTCTTCCCG

To evaluate their ability to take up tFNAs and ssDNAs, HaCaT and HSF cells were treated with Cy5-tFNAs (125 nM) or Cy5-ssDNAs (125 nM). We clearly observed that the fluorescence of Cy5 after exposure to tFNAs was visibly stronger than that of Cy5-ssDNAs after 10 h of treatment (Fig. 1f, g). The results revealed that tFNAs could easily be taken up by these two cell lines, while naked oligodeoxynucleotides were not absorbed in abundance.

### tFNAs promoted cellular proliferation by modulating cell cycles

**Fig. 2:**
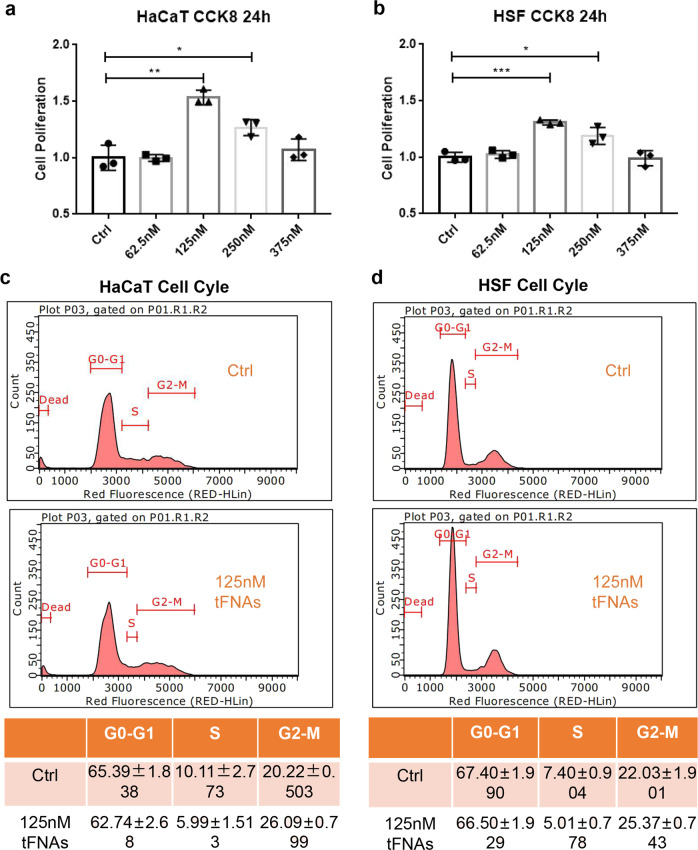
The effects of tFNAs on cell proliferation and cell-cycle regulation. **a** CCK-8 results of HaCaT cells after treatment with different concentrations of tFNAs for 24 h. Data are presented as the mean ± SD (*n* = 3). **b** CCK-8 results of HSF cells after treatment with different concentrations of tFNAs for 24 h. Data are presented as the mean ± SD (*n* = 3). **c** Flow-cytometry results of the cell cycle of HaCaT cells treated with or without 125 nM tFNAs for 24 h. Data are presented as the mean ± SD (*n* = 3). **d** Flow-cytometry results of the cell cycle of HSF cells treated with or without 125 nM tFNAs for 24 h. Data are presented as the mean ± SD (*n* = 3). Significance: *p < 0.05, **p < 0.001, ***p < 0.001

To determine the effect that tFNAs have on cell proliferation, CCK-8 and cell-cycle assays were performed. It is clear that after treatment with tFNAs, both HaCaT and HSF cells had greater proliferation ability than that of the control cells (Fig. 2a, b). The outcome of the CCK-8 treatment also showed that a concentration of 125 nM is most suitable for promoting self-renewal in both cell types; as such, we used 125 nM tFNAs for subsequent cell-cycle assays. Flow cytometry was performed after 24 h of treatment to further explore the changes in cell cycling in HaCaT and HSF cells. According to the cell-cycle assays, the number of cells in the proliferative phase (G2-M phase) was significantly increased, and the number of cells in the synthesis phase (S phase) was decreased (Fig. 2c, d). These outcomes suggest that 125 nM tFNAs could promote cell mitosis and cause it to proceed from the S phase to the G2/M phase. Taken together, these results show that tFNAs could promote the proliferation of HaCaT and HSF cells by modulating cell-cycle progression.

### tFNAs increased cellular migration

**Fig. 3:**
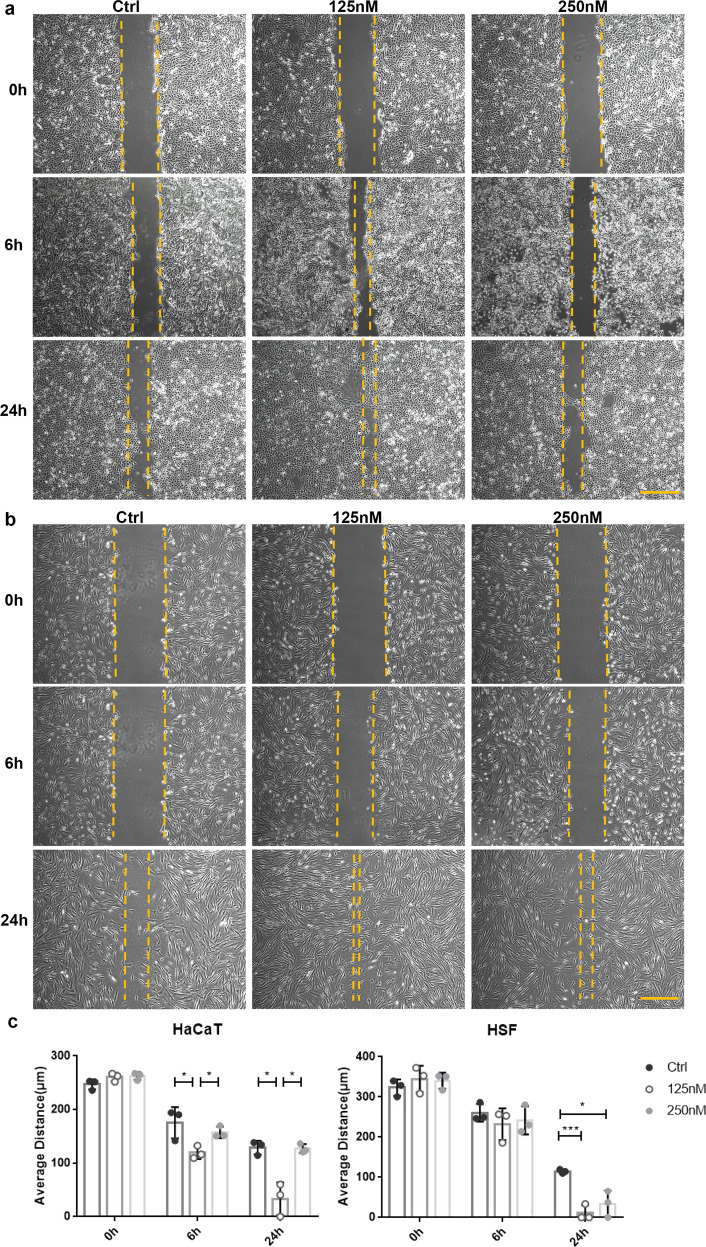
The effects of tFNAs on cell migration. **a** Images of scratch tests on HaCaT cells treated with different concentrations of tFNAs at 0 h, 6 h, and 24 h. Scale bars are 200 μm. **b** Images of scratch tests on HSF cells treated with different concentrations of tFNAs at 0 h, 6 h, and 24 h. Scale bars are 200 μm. **c** Statistical analysis of scratch tests. Data are presented as the mean ± SD (*n* = 3). Significance: *p < 0.05, ***p < 0.001

In this study, scratch experiments were used to determine whether tFNAs influenced cell migration ability. It is apparent that tFNA treatment promoted cellular migration to cover the scratches (Fig. 3a, b). As exhibited, both HaCaT and HSF cells that were treated with 125 nM tFNAs had the strongest migration ability among the three groups (Fig. 3c), in accordance with the CCK-8 assay results. Thus, the concentration of tFNAs used in all subsequent experiments was 125 nM.

### tFNAs increased the anti-inflammatory reaction and cell secretion by activating the AKT-signaling pathway

**Fig. 4:**
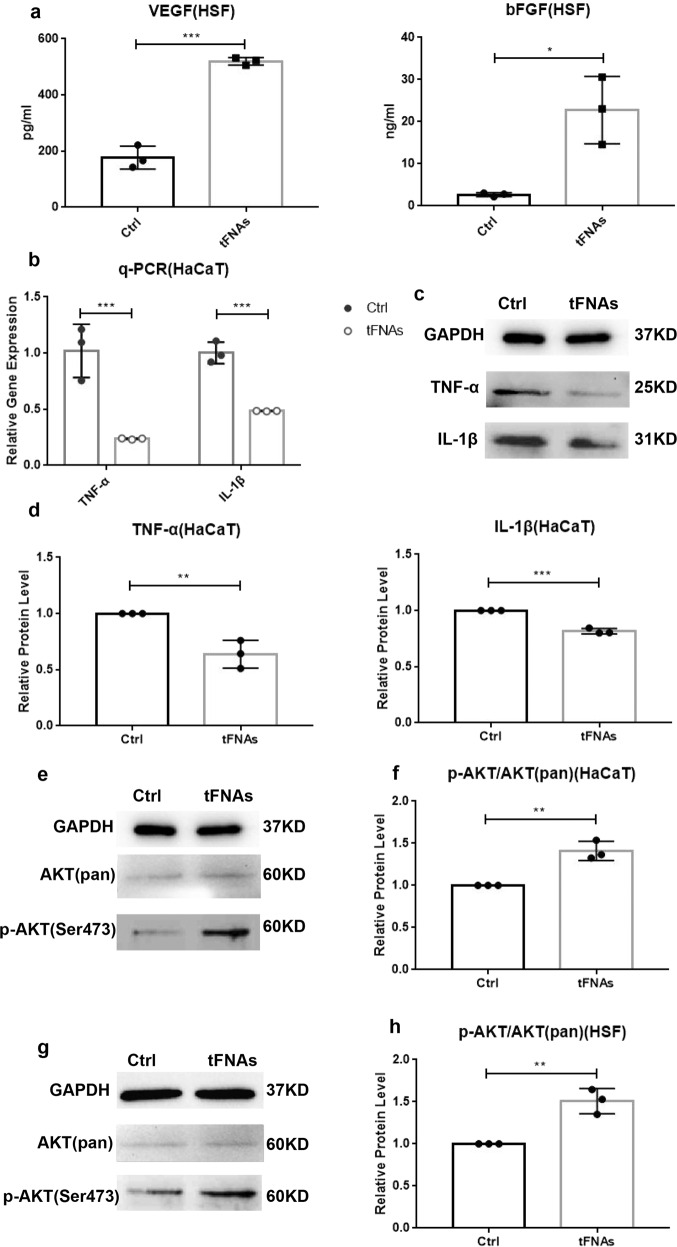
The effects of tFNAs on growth factors, inflammatory cytokines, and the AKT-signaling pathway. **a** ELISA results of VEGF and bFGF secretion after 24 h of treatment with 0 or 125 nM tFNAs. Data are presented as the mean ± SD (*n* = 3). **b** Relative gene expression levels of TNF-α and IL-1β after HaCaT cells were exposed to 0 or 125 nM tFNAs for 24 h. Data are presented as the mean ± SD (*n* = 3). **c** Detection of TNF-α and IL-1β protein expression by western blot analysis of HaCaT cells treated with or without 125 nM tFNAs for 24 h. **d** Semiquantitative analysis of TNF-α and IL-1β protein expression. Data are presented as the mean ± SD (*n* = 3). **e** Protein levels of AKT (pan) and p-AKT (Ser473) in HaCaT cells treated with or without tFNAs. **f** Quantification of the phosphorylation level of AKT in HaCaT cells. Data are presented as the mean ± SD (*n* = 3). **g** Protein expression of AKT (pan) and p-AKT (Ser473) in HSF cells treated with or without tFNAs. **h** Quantification of the phosphorylation level of AKT in HSF cells. Data are presented as the mean ± SD. Significance: *p < 0.05, **p < 0.01, ***p < 0.001

An enzyme-linked immunosorbent assay (ELISA) was used to test the secretion of vascular endothelial growth factor (VEGF) and basic fibroblast growth factor (bFGF) by HSF cells. The ELISA results indicated that when cultured with 125 nM tFNAs, the secretion levels of both VEGF and bFGF increased remarkably in HSF cells (Fig. 4a). The protein expression levels of tumor necrosis factor-alpha (TNF-α), interleukin-1 beta (IL-1β), AKT (pan), and p-AKT (Ser473) were examined using western blotting, and qPCR was used to evaluate the gene expression levels of TNF-α and IL-1β. The outcome revealed that after treatment with 125 nM tFNAs, the protein levels of TNF-α and IL-1β in HaCaT cells evidently decreased as did the gene expression, which indicates that tFNAs reduced the inflammatory reaction of keratinocytes (HaCaT cells) (Fig. 4b–d). In addition, at a fixed level of GAPDH, the p-AKT/AKT(pan) ratio in both HaCaT and HSF cells, was increased by tFNA treatment (Fig. 4e–h). This revealed that the phosphorylation level of the AKT pathway was increased after exposure to tFNAs. Therefore, we can conclude that tFNAs are able to promote growth factor secretion by fibroblasts and inhibit the inflammatory response of keratinocytes by activating the AKT-signaling pathway.

### tFNAs accelerated cutaneous wound closure and decreased scar formation in vivo

**Fig. 5:**
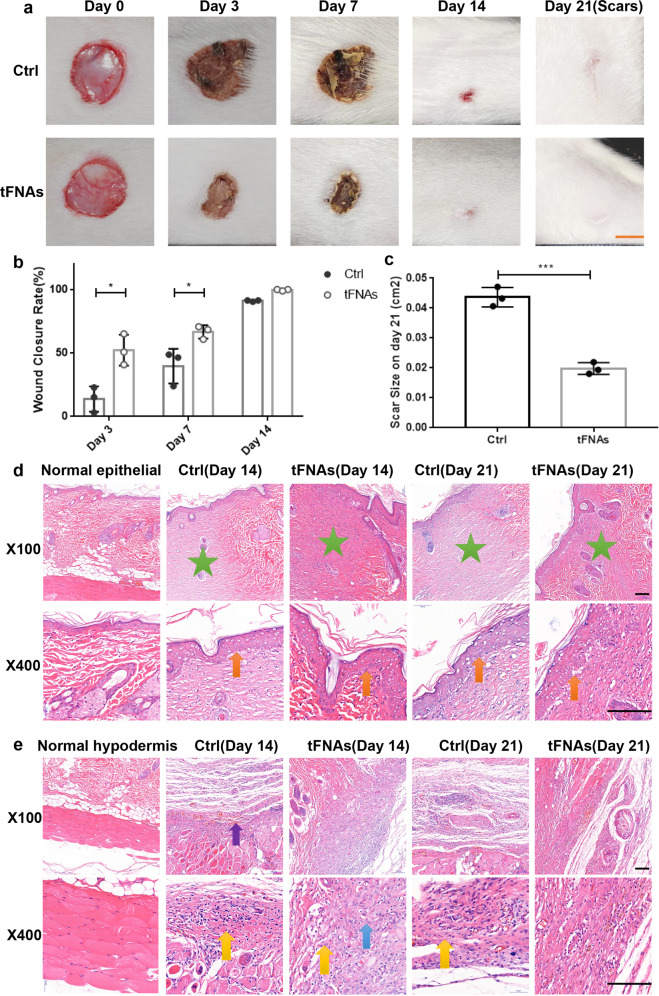
The effects of tFNAs on wound healing and scarring in rats. **a** Photographs of skin wounds on rats treated with saline or 125 nM tFNAs at different times (0, 3, 7, 14, and 21 days after surgery). Scale bars are 5 mm. **b** Comparison of the wound healing rate between the saline and tFNA groups. Data are presented as the mean ± SD (*n* = 3). **c** Size of scars measured on the 21st day postwounding. Data are presented as the mean ± SD (*n* = 3). **d** H&E staining of the epidermis (green star: scar area; orange arrow: epithelium thickening) Scale bars are 100 μm. **e** H&E staining of the hypodermis (yellow arrow: inflammatory cell infiltration; blue arrow: proliferation of fibroblasts; purple arrow: dermal hemorrhage) Scale bars are 100 μm. Significance: *p < 0.05, ***p < 0.001

To explore whether tFNAs can aid the wound healing process in vivo, skin wound models were created with rats, and then equal amounts of saline or 125 nM tFNAs were injected into the surrounding area. Compared with the control group, the tFNA group exhibited a markedly advanced wound healing rate (Fig. 5a, b). On the 21st day after surgery, when all wounds in both groups were healed, rats from the tFNA group had visibly smaller scars than those from the control group (Fig. 5a, c). In addition, the wound sites were analyzed histopathologically on days 14 and 21 to estimate the quality of wound closure. Hematoxylin and eosin (H&E) staining of the epithelial tissue indicated that in the tFNA group, the scar areas were smaller, and the epidermis grew thicker than in the saline group (Fig. 5d). In addition, H&E staining of the hypodermis suggested that inflammatory cell infiltration in the tFNA group was less than that in the control group (Fig. 5d). These results indicate that tFNAs can promote skin wound healing and reduce scarring.

### tFNAs alleviate fibrosis and inflammatory reaction in scar areas

**Fig. 6:**
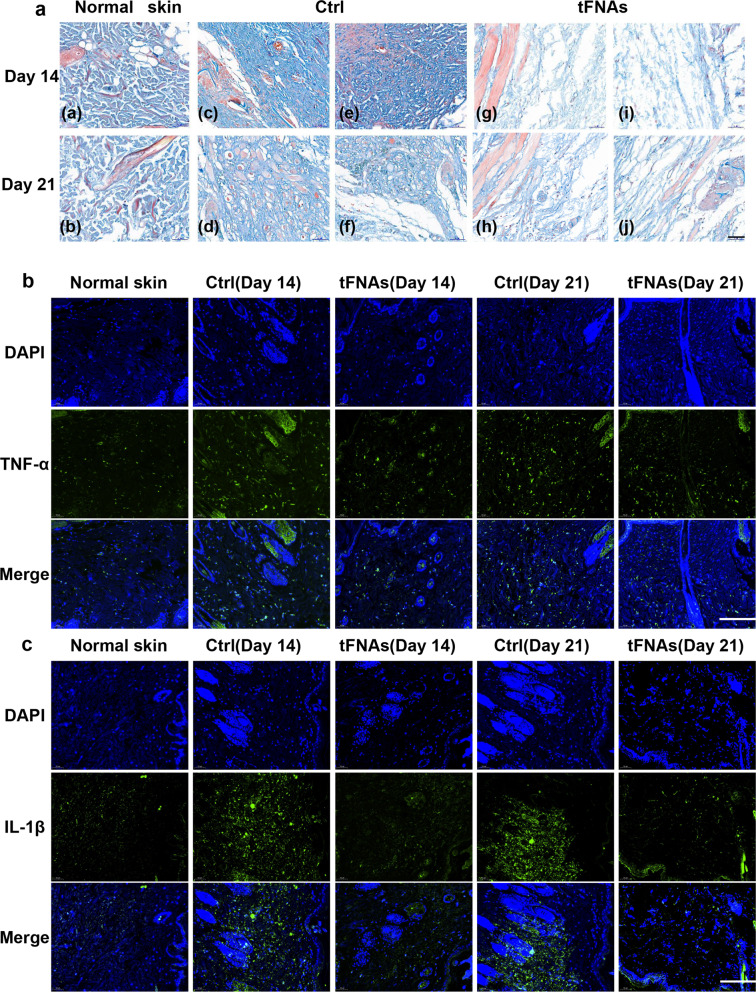
Antifibrotic and anti-inflammatory effects of tFNAs. **a** Masson staining of normal skin and skin samples from the control group and the tFNA group. Scale bars are 50 μm. **b** Immunofluorescence staining of TNF-α protein expression in normal skin and skin samples from the control group and the tFNA-treated group. Scale bars are 300 μm. **c** Immunofluorescence staining of IL-1β expression in normal skin and skin samples from the control group and the tFNA-treated group. Scale bars are 300 μm

Masson staining clearly showed that fibrosis occurred in both the control and tFNA-treated groups owing to the surgery. However, skin fibrosis was obviously mitigated in wounds treated with 125 nM tFNAs compared with those treated with saline (Fig. 6a and Table 2). Additionally, immunofluorescence staining of the skin showed that the protein levels of TNF-α and IL-1β decreased significantly in wounds treated with tFNAs compared with those treated with saline (Fig. 6b, c). This suggests that the anti-inflammatory effects of tFNAs also occured in our in vivo models. In summary, tFNAs helped alleviate fibrosis and reduce inflammatory reactions during the wound healing process in vivo.

**Table 2:** Percentage of collagen fibrosis in skin samples (%)

Letter	View 1	View 2	View 3	Average fibrosis rate
a	63.21	68.62	63.85	65.23
b	54.37	71.20	69.77	65.11
c	87.92	86.07	90.41	88.13
d	84.53	87.03	88.64	86.74
e	92.11	92.25	91.46	91.94
f	90.79	90.86	90.18	90.61
g	79.87	71.98	85.19	79.01
h	83.45	80.73	76.49	80.22
i	70.73	81.22	81.51	77.82
j	81.68	74.95	72.61	76.42

## Discussion

Every day, thousands of people are wounded for different reasons. The wound healing process may be painful, and there is also a risk of infection in wounds that are difficult to heal. A poorly healed wound can also become a scar, affecting the patient’s physiological function and mental health. Shortening the duration of wound healing and reducing scarring are complex problems that many scientists are eager to solve. Thus, we sought to explore the effects of tFNAs on various stages of the wound healing process.

As previously suggested, tFNAs can be taken up quickly by cells via caveolin-mediated endocytosis, while the uptake of ssDNAs into those same cells is minimal.^23–25^ Moreover, cellular uptake is the key to the various effects of tFNAs on wound healing.^26,28^ Therefore, after we successfully prepared tFNA nanostructures of an appropriate size and with a stable zeta potential, we demonstrated that tFNAs could be taken up in abundance by these two types of cells.

Previous studies have indicated that tFNAs can promote the proliferation of various cell types.^24–27,29,34,35^ Therefore, in this research, we aimed to determine whether this type of effect also existed in HaCaT and HSF cells. CCK-8 assays were used to examine cell proliferation, while cell-cycle assays were used to analyze changes in the number of cells at each stage. The observed changes in the cell cycle provided support for the results of the CCK-8 assays.

As shown in previous studies, tFNAs are able to promote cell migration.^27–29,35^ Scratch experiments are widely performed to investigate cell migration in vitro, but there are still factors that can interfere with these outcomes. In this study, to minimize variability, all scratches were made by the same person. In addition, the scratch areas of three random fields of view were measured and then averaged for all time points in all treatments.

On the basis of former studies, over the course of skin wound healing, fibroblasts secrete a series of growth factors that help to regulate multiple effects associated with cell proliferation, migration, and cycling as peptide mediators.^32,33^ VEGF and bFGF are both crucial among the various pro-angiogenic mediators involved in the healing of cutaneous wounds.^36–39^ At the same time, the inflammatory phase is essential for homeostasis maintenance during cutaneous wound healing.^1–4^ Inflammatory cytokines, including TNF-α and IL-1β, are of vital importance during this state.^40–42^ To encourage the healing of skin wounds and lessen scar formation, it is necessary to increase the secretion of fibroblasts and alleviate the inflammatory response. Previous studies have reported the anti-inflammatory effects of tFNAs in macrophages.^30^ In this research, we demonstrated that tFNAs are able to increase the secretion of VEGF and bFGF while decreasing the production of TNF-α and IL-1β.

After being taken in, tFNAs can act on different signaling pathways, resulting in a series of biological effects. The AKT-signaling pathway is widespread in the human body and plays a decisive role in various physiological processes including skin wound healing.^43^ Many scientists believe that the activation of the AKT-signaling pathway leads to enhanced skin wound healing.^44–48^ To explore the mechanisms by which tFNAs elicit their effects in vitro, we investigated the changes in the AKT pathway in HaCaT and HSF cells. The observed increase in AKT phosphorylation indicated that the activation of the AKT-signaling pathway was related to the cellular effects of tFNAs.

To investigate the influences of tFNAs on the healing process of animal skin wounds, Sprague–Dawley (SD) rats were used to establish in vivo animal models. For an autologous control, we created two wounds on the back of each rat. Saline was injected into the area surrounding the left wound, while an equal volume of 125 nM tFNA was injected into the area surrounding the right wound. When treated with tFNAs, the rats not only showed more rapid wound healing than those in the control group but also had less scarring after the wounds healed. The results of H&E staining showed that there was less inflammatory infiltration in the tFNA group than in the control group. There were two cases of dermal hemorrhaging in the control group but none in the experimental group, which may suggest the protective effects of tFNAs on the vascular endothelium. The hyperplasia of fibroblasts and an increase in re-epithelialization were also observed. These results are consistent with those of our in vitro experiments.

Skin fibrosis and inflammatory reactions are considered to play major roles in hypertrophic scarring after cutaneous wound healing.^3,4^ The focus of research to reduce scar formation is to reduce skin fibrosis and inflammatory reactions. The Masson staining results showed that although the surgical process led to skin fibrosis, the application of tFNAs reduced the rate of skin fibrosis. In addition, the results of the immunofluorescence studies showed increased production of TNF-α and IL-1β on days 14 and 21 in the saline group compared with the tFNA group, while the levels of TNF-α and IL-1β decreased significantly after the application of tFNAs. This suggests that the anti-inflammatory effects of tFNAs observed in vitro also applied to the in vivo models. The decrease in the rate of skin fibrosis and the reduction in inflammation may be important reasons for the reduced scarring in the tFNA group.

## Conclusions

In conclusion, this is the first study using biological nanophase materials composed of nucleic acids to enhance cutaneous wound healing in both in vitro studies and animal models. tFNAs have the ability to promote HaCaT and HSF cell migration and proliferation; they can increase the secretion of growth factors in HSF cells and relieve inflammatory reactions in HaCaT cells by activating the AKT-signaling pathway in vitro. Additionally, tFNAs are able to provoke skin wound healing and reduce scar formation in vivo. To the best of our knowledge, these are the first published results showing the regenerative effects of tFNAs on skin. We believe that tFNAs have great potential for clinical application in the acceleration of wound healing and reduction of scar formation.

## Experimental section

### Synthesizing tFNAs

Four different ssDNAs were used to construct the tFNAs. To do so, the ssDNAs were dissolved in equal proportions in a well-mixed TM buffer (pH 8.0) containing 10 mM Tris-HCl and 50 mM MgCl_2_. The mixture was then denatured at 95 °C for 10 min, followed by a rapid cooling period at 4 °C for at least 30 min.

### Cell culture

The HaCaT and HSF cell lines were purchased from Mingjing Biology (Shanghai, China), and the cells were cultured in high-glucose Dulbecco’s modified Eagle’s medium (H-DMEM) mixed with 10% (v/v) fetal bovine serum (FBS) and 1% (v/v) penicillin-streptomycin in an incubator with a controlled environment at 37 °C and 5% CO_2_. The cell culture medium was changed twice per week.

### Characteristics of tFNAs

We used HPCE and TEM to confirm that tFNAs were successfully synthesized. The size and zeta potential of tFNAs were determined using a Zetasizer Nano ZS90 (Malvern Instrument Ltd., Malvern, UK).

### Cellular uptake of Cy5-loaded tFNAs

First, 1 × 10^5^ cells were seeded into confocal dishes and cultured in standard medium overnight. Fresh H-DMEM with only 1% FBS was used thereafter, with the addition of Cy5-loaded tFNAs (125 nM) or Cy5-loaded ssDNAs (125 nM). After 10 h of treatment, 4% (w/v) paraformaldehyde solution (Boster, Wuhan, China) was applied to fix the cell samples for at least 30 min. FITC-labeled phalloidin and DAPI(4′6-diamidino-2-phenylindole), purchased from Sigma (St. Louis, MO), were then used to stain the cytoskeleton and nucleus of each cell. The cytoskeleton was stained for 1 h and the nucleus was stained for 10 min. All of the samples were washed with phosphate-buffered saline (PBS) three times after each step. Finally, a confocal laser microscope (Nikon N-SIM, Tokyo, Japan) was used to observe the stained samples.

### Proliferation assay

CCK-8 and cell-cycle-assay kits(KeyGEN, Jiangsu, China) were used to assess the proliferation of the cells. For CCK-8, a 96-well plate was used with approximately 8000 cells per well. After the cells were cultured overnight, the medium was replaced with H-DMEM with 1%(v/v) FBS and tFNAs at different concentrations. For the control group, the cell medium contained no tFNAs. Cell proliferation was examined after 24 h of treatment with CCK-8 solution. A flow-cytometry assay was used to examine the cell cycle. After 24 h of treatment, samples were collected with 0.25% (w/v) trypsin-EDTA solution and fixed with 70% ethanol at 4 °C for 12 h. After rewashing with PBS, the samples were incubated with 50 μL of RNase at 37 °C for 30 min. Next, 450 μL of propidium iodide (PI) solution was added to samples for 30 min at 4 °C in the dark. The percentages of cells in the G0-G1, S, and G2-M stages were then measured using a Millipore Guava easyCyte HT (Burladingen, Germany). The changes in the cell-cycle distribution were investigated using FlowJo software.

### Migration assay

Scratch experiments were used to investigate cell migration behavior. Cells (2 × 10^5^) were plated in a 35 mm diameter dish and cultured overnight. After washing with PBS, a pipette tip was used to create a cross-shaped scratch in each well. The cells were rewashed three times and cultured with H-DMEM with 1% (v/v) FBS containing different concentrations of tFNAs (0, 125, and 250 nM) for 24 h. Images of the samples were taken after 6 and 24 h of treatment. The widths of the scratches were measured, recorded, and then compared with the original scratches at 0 h using Image-Pro Plus software (Medical Cybernetics, USA).

### Western blotting

The protein expression of inflammatory factors (TNF-α and IL-1β), AKT (pan) and p-AKT (Ser473) were examined by western blotting. Six-well plates were used to culture cells. After culturing overnight, the cells were treated with low-FBS medium containing 125 nM tFNAs or no tFNAs for 24 h. The total cellular protein was collected using whole-protein extraction kits (KeyGen). The mixtures containing protein samples and quarter volumes of 5× loading buffer were boiled for 20 min. SDS-PAGE gels (10 and 12%) were applied to separate the target proteins. After transferring the proteins onto PVDF membranes, 5% bovine serum albumin(BSA) or skim milk was used to block the membranes for 1 h. The membranes were then incubated with primary antibodies at 4 °C overnight. After the membranes were washed three times with Tris-buffered saline and Tween 20(TBST), the membranes were incubated with secondary antibodies(Beyotime, Shanghai, China) for 1 h. After the membranes were washed again, the proteins were detected using a Bio-Rad (Hercules, CA, USA) detection system. Considering the stable expression of glyceraldehyde-3-phosphate dehydrogenase(GAPDH) in both HaCaT and HSF cells, GAPDH expression was used as the internal control for both cell lines. The anti-TNF-α (ab66579) and anti-IL-1β (ab2105) antibodies were purchased from Abcam (Cambridge, UK). The anti-GAPDH (5174), anti-AKT (pan) (2920), and anti-p-AKT(Ser473) (4060) antibodies were purchased from Cell Signaling Technology (Boston, USA).

### Quantitative PCR assay

Gene expression of TNF-α and IL-1β in HaCaT cells was examined using quantitative PCR (qPCR). qPCR assays were performed with a PrimeScript RT-PCR kit (Takara, Dalian, China). TRIzol (Thermo Fisher Scientific, MA, USA) was used to extract the total cell RNA. The target cDNA was amplified using a 96-well QuantStudio 3 Real-Time PCR system (Thermo Fisher, Chengdu, China) at 95 °C for 30 s followed by 40 cycles of 95 °C for 5 s and 60 °C for 34 s. The final volume of complementary DNA (cDNA) prepared with a cDNA synthesis kit (Takara, Dalian, China) was 20 μL. The internal control used in these experiments was GAPDH. Melting curves were produced for each reaction to detect incorrect priming and primer dimer formation. The primer sequences were as follows: TNF-α(forward: 5**′**-CCTGCCCCAATCCCTTTATT, reverse: 5**′**-CCCTAAGCCCCCAATTCTCT); IL-1β(forward: 5**′**-ACAACAGGAAAGTCCAGGCTA, reverse: 5**′**-TGGCAGAAAGGGAACAGAA); and GAPDH (forward: 5**′**-TCATGACCACAGTCCATGCCATCA, reverse: 5**′**-CCCTGTTGCTGTAGCCAAATTCGT). The 2 − ΔΔCT method was used to analyze the PCR results.^49^

### ELISA

VEGF and bFGF secretion from HSF cells was analyzed using ELISA kits (Meimian Biology, Wuhan, China). Cells were seeded in six-well plates incubated overnight, and then treated with H-DMEM with 1% (v/v) FBS containing 125 nM tFNAs or no tFNAs. After 24 h of treatment, the supernatants were collected and subjected to ELISA according to the manufacturer’s protocol for qualitative assessment.

### Animal models

Female SD rats (*n* = 6) weighing 250–280 g were purchased from DossyLife Science (Chengdu, China). The rats were kept individually in an environment with a 12/12-h light/dark cycle. After successful anesthetization by pentobarbital sodium injection (20 mg/kg) into the abdominal cavity, the rats were shaved and two round samples of full thickness skin were collected by a 10-mm punch biopsy, with one sample from the left and one from the right side of the rat’s back. After surgery, the surrounding areas of the right wounds were administered a subcutaneous injection of 100 μL of 125 nM tFNAs (dissolved in saline) once a day for the next seven days. An equal amount of saline was administered to the wound on the left side using the same procedure. The wound areas were evaluated and recorded at days 0, 3, 7, 14, and 21. Samples of skin tissue were collected for further study after the test rats were sacrificed. Wound closure rate (%) = (1 − wound size on day X /original wound size on day 0) × 100%.

### Histological analysis

Samples of skin tissue from both the control and tFNA groups were collected on the 14th and 21st days after the surgery. Additionally, normal skin samples were collected for comparison. After fixation with a 4% paraformaldehyde solution, the samples were dehydrated and paraffin-embedded and then sliced into 4 μm sections. H&E staining and Masson staining were performed on the tissue slides. The tissue slides were imaged using an FSX100 microscope (Olympus, Tokyo, Japan). After staining, all samples were analyzed in random order on the same day. Three different visual fields for each slide were measured and averaged to acquire the epidermal thickness and fiber percentage of each sample.

### Immunofluorescence staining

To visualize the transformation of TNF-α and IL-1β expression in vivo, slides were incubated overnight with anti-TNF-α (ab66579) and anti-IL-1β (ab2105) antibodies. After washing three times with PBS, the slides were incubated with secondary antibodies for 1 h. The slides were then exposed to DAPI for 10 min to stain the nuclei. Finally, sections were observed using fluorescence microscopy.

### Statistical analysis

All presented data came from at least three separate experiments and are reported as the mean ± standard deviation (SD). GraphPad Prism 7.0 software was used for statistical analysis. To analyze the experimental data, one-way ANOVA together with Student’s *t*-test were used to determine the significant difference between samples. *p < 0.05, **p < 0.01, and ***p < 0.001 were used as thresholds for statistical significance.

## Data Availability

Additional data collected during this study are available from the corresponding author upon reasonable request.
